# Two Cases With Type 1 Diabetes Treated With Insulin Pump Therapy Using a Telemedicine Approach During the COVID-19 Pandemic

**DOI:** 10.7759/cureus.51607

**Published:** 2024-01-03

**Authors:** Haremaru Kubo, Kazuhiro Sugimoto

**Affiliations:** 1 Diabetes Center, Ohta Nishinouchi Hospital, Koriyama, JPN; 2 Department of Diabetes and Metabolism, Tohoku University Hospital, Sendai, JPN

**Keywords:** covid-19, telemedicine, intermittently scanned continuous glucose monitoring, insulin pump, type 1 diabetes

## Abstract

Type 1 diabetes mellitus (T1D) is an autoimmune-related disease resulting in insulin dependency, treated with insulin injection via pen devices or continuous subcutaneous insulin infusion (CSII). Face-to-face instruction for managing insulin injection and dosing and machine-to-device troubleshooting are required early to initiate CSII from insulin injections. Thus, T1D individuals may encounter significant barriers to pen devices or CSII introduction if they live in remote rural areas. In this regard, intermittently scanned continuous glucose monitoring (isCGM) can share visualized glucose profiles via a cloud-platform-based system, offering the potential as an effective tool in telemedicine. Herewith, we report two cases of subjects with T1D living in remote rural areas whose CSII was safely introduced in outpatient settings with the aid of cloud-platform-based isCGM and a video-meeting tool. They showed improved glucose profiles after CSII initiation. Even under the coronavirus disease 2019 (COVID-19) pandemic, the telemedicine system enabled healthcare providers to monitor glucose profiles and confirm device procedures of CSII. We emphasize the usefulness of online instruction with cloud-platform-based isCGM for introducing CSII in cases with barriers to healthcare access, particularly during the COVID-19 pandemic.

## Introduction

Type 1 diabetes mellitus (T1D) is a disease mediated by autoimmune triggers destroying pancreatic beta cells with exogenous insulin dependency, the cause of which is still unclear. Generally, there are two methods of exogenous insulin supplementation: multiple daily injections (MDI) using pen devices and continuous subcutaneous insulin infusion (CSII) [1] using insulin pumps. In MDI, basal insulin for modulating fasting blood glucose and short-acting insulin for modulating postprandial blood glucose were administered via pen devices several times a day. However, insulin-dose adjustments have limited flexibility in MDI because once injected, basal insulin cannot be adjusted for diurnal glucose changes, such as due to the dawn phenomenon [2] and during or after exercise. Otherwise, with CSII, basal insulin can be set as "units per hour" anytime daily. Thus, based on the guidelines, CSII is recommended [2] as the first therapeutic option for persons with T1D, and unstable glucose levels could be improved with CSII with precise and personalized dosing. An automated insulin delivery system (AID) [3] also enables automatically modulated insulin doses in response to glucose levels, though it is more expensive than CSII.

Otherwise, the insulin pump introduction has several limitations, including healthcare access for frequent outpatient visits, mainly in early-stage for accustoming mechanical troubles and economic problems in the countries where CSII is self-funded [4]. Rural people in Japan have many difficulties with healthcare access because Japan is a mountain- or island-rich country, and approximately 10% of the population lives in hard-to-move areas [5]. Therefore, they could not attend to outpatients frequently and felt demotivated to introduce CSII by their circumstances. Thus, in Japan, the initiation of CSII has generally been performed on an inpatient basis for sufficient education and training in emergencies, unlike abroad [6].

Intermittently scanned continuous glucose monitoring (isCGM) is a new technology for glucose management [7], displaying trends in glucose profiles. Increased or decreased glucose levels are indicated via time-above range (TAR; glucose levels >180 mg/dL), time-in-range (TIR; glucose levels 70-180 mg/dL), and time-below-range (TBR; glucose levels <70 mg/dL). Although hemoglobin A1c (HbA1c) is still the gold standard for assessing glucose control, these parameters have recently attracted attention as novel indicators, leading to meaningful benefits in the quality of life and personalized glycemic management [7]. In addition, the recent-implemented cloud-platform-based system Libre View® (Abbott Diabetes Care Inc., CA, USA) enables persons with diabetes, their families, and healthcare professionals to access glucose trends online for remote diabetes management even under the coronavirus disease 2019 (COVID-19) pandemic [8]. This cloud-based system provides a contactless share of glucose levels to healthcare providers and outpatient subjects. Moreover, recent advances in video-meeting tools enable interactive conversations via the Internet between healthcare providers and subjects in outpatient settings. Otherwise, although there are some reports about the usefulness of telemedicine in T1D subjects from the viewpoint of improved medical expenses and treatment motivation [9] or glucose profiles [10] from abroad, only a few reports show the usefulness of telemedicine for the subjects with diabetes in Japan, such as a report about type 2 diabetes [11] and there is no previous report using these telemedicine systems for CSII introduction as far as we know. Herein, we report the first case report using telemedicine systems to introduce CSII in the COVID-19 pandemic.

## Case presentation

Case 1

**Figure 1:**
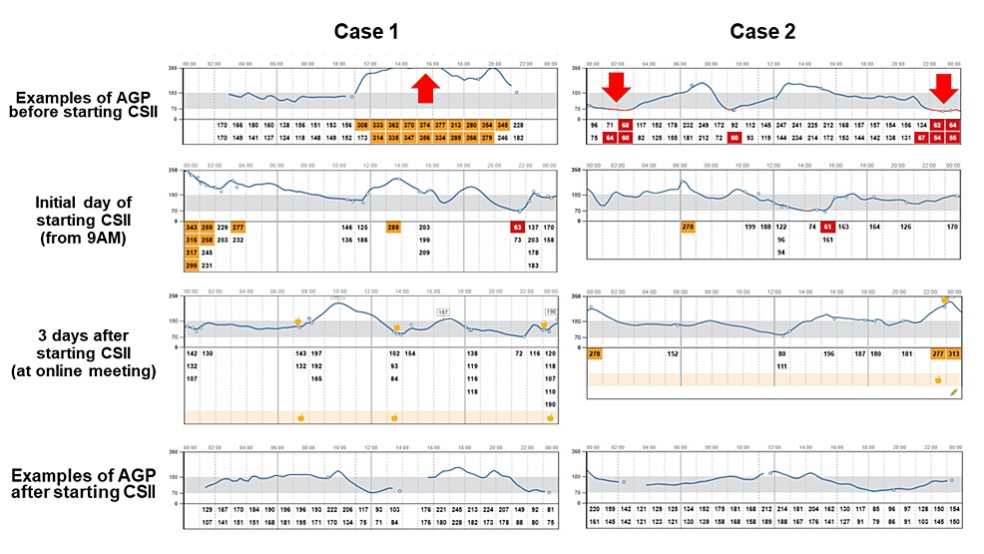
Examples of glucose profiles in both cases Examples of glucose profiles on isCGM of cases 1 and 2 are shown. Arrows are the typical timing, significantly increased or decreased glucose fluctuation. isCGM - intermittently scanned continuous glucose monitoring; AGP - ambulatory glucose profile; CSII - continuous subcutaneous insulin infusion

A 36-year-old man with an eight-year history of T1D living approximately 1.5 hours by car from our hospital had been treated with basal-bolus MDI (insulin to carbohydrate ratio [CIR], 1 U:10-10-10 g; insulin sensitivity factor [ISF], 60 mg/dL/unit; target glucose levels <120 mg/dL) with insulin lispro and degludec 12-14 U. His endogenous insulin secretion was decreased (when the fasting plasma glucose [FPG] was 120 mg/dL, the fasting C-peptide immunoreactivity [CPR] and immunoreactive insulin [IRI] was 0.3 ng/dL and 0.6 mU/mL), and anti-glutamic acid decarboxylase (GAD) antibodies were positive (279.1 U/mL). His HbA1c level was >10.0% for several years and remained above the target level (<7.0%) shown in Japanese guidelines [1] or the American and European diabetes association [2] before the initiation of CSII. He used isCGM for four years to manage hypoglycemia, mainly due to excessive physical activities carrying heavy merchandise during his work at a drugstore. Owing to the fear of unexpected hypoglycemia, he was willing to maintain high blood glucose levels during the daytime (Figure 1), which resulted in more elevated glucose levels with TAR and TIR of 74% and 23% than the recommended percentage (<25%, >70%) in consensus statements [2,12], respectively (Table 1).

**Table 1:** Summary of laboratory data and isCGM indicators before and after CSII initiation After CSII initiation, all glucose-related parameters improved. In particular, the TIR and TBR were increased and decreased, respectively. CSII - continuous subcutaneous insulin injection; HbA1c - hemoglobin A1c; TAR - time-above range; TIR - time-in range; TBR - time-below range; %CV - percent coefficient of variation

Case	Laboratory data	Before CSII	One month after CSII	Four months after CSII
Case 1	Mean glucose level (mg/dL)	250	177	205
	HbA1c	10.5	8	7.9
	TAR (%)	74	47	52
	TIR (%)	23	51	47
	TBR (%)	3	2	1
	%CV	40.8	37.3	40.5
Case 2	Mean glucose level (mg/dL)	172	193	163
	HbA1c	7.9	7.9	7.1
	TAR (%)	40	49	37
	TIR (%)	54	50	60
	TBR (%)	6	1	3
	%CV	40.6	39.4	34.5

Case 2

A 46-year-old woman with an 11-year history of T1D living approximately one hour by car from our hospital had been treated with basal-bolus MDI (CIR 1 U: 10-10-10 g; ISF of 50 mg/dL/unit; target glucose levels <120 mg/dL) with insulin lispro and glargine 12 U. Her endogenous insulin secretion was almost under detection limit (when the FPG was 149 mg/dL, the fasting CPR and IRI was <0.02 ng/dL and 0.6 mU/mL), and anti-GAD antibodies were positive (141.3 U/mL). Her HbA1c level was around 7-8% for several years. Notably, she often experienced hypoglycemia as an increased TBR of approximately 6-8% before the initiation of CSII using isCGM for several years. Especially, unexpected hypoglycemia occurred mainly late at night and sometimes in the daytime due to increased physical activity while at work in a factory (Figure 1) with TAR, TIR, and TBR of 40%, 54%, and 6%, respectively (Table 1).

**Figure 2:**
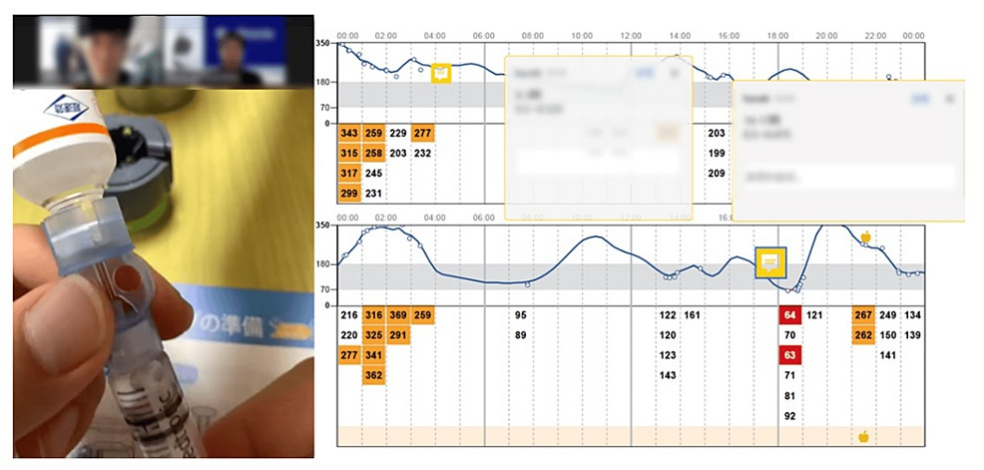
The telemedicine image with video-meeting tools sharing isCGM data and procedure Desktop image of telemedicine in the present case. We confirmed the insulin pump procedure (left side) through a video meeting tool (Zoom Video Productions Inc, San Jose, CA) and glucose trends using LibreView® (Abbott Diabetes Care Inc., CA, USA), which is displayed on the right side. A video-cropped image is obtained at the time of exchanging insulin syringes. isCGM - intermittently scanned continuous glucose monitoring

Based on the circumstances above, we proposed the usage of CSII for better glycemic management, although AID was refused for economic reasons. They also refused hospitalization, a typical CSII introduction course in Japan, for the initial adjustment of the CSII owing to the fear of infection during the COVID-19 pandemic and the long-distance traveling difficulties. Therefore, we proposed an alternative plan as CSII with isCGM. First, we assessed the subjects' patterns of glucose trends before CSII initiation via isCGM (Figure 1). After that, according to these trends, we initiated CSII using MiniMed^TM^ 670G (Medtronic, Northridge, CA, USA) in both of the cases with insulin lispro at the basal insulin rate of 0.5 U/hour for 24 h (case 1: presented flat glucose trend) and 0.45 U/hour at 11 PM-1 AM, 0.50 U/hour at 1 AM-5 AM, 0.45 U/hour at 5 AM-7 AM, 0.50 U/hour at 7 AM-11 PM (case 2: presented decreased glucose levels mainly late at night) with same CIR and ISF before initiation of CSII over isCGM. Subsequently, we offered him an online meeting (Zoom Video Productions Inc., San Jose, CA) three days after the face-to-face CSII initiation to confirm glucose trend via isCGM and any troubleshooting or insulin-loading procedure when needed to exchange insulin syringes and other supplies. During the meeting, we observed the actual procedure of filling an insulin reservoir and inserting an infusion set (Figure 2), which was appropriate and safe. Therefore, although we initially planned another online procedure checkup, we did not conduct an online follow-up. Concurrently, we discussed the glucose trends and adjusted the bolus insulin settings and basal insulin rate based on data from the cloud-platform-based system LibreView® (Figure 1). Though both cases showed hypoglycemia on the first day of CSII initiation, they showed relatively well-controlled glucose trends (Figure 1). Based on these data we proposed correction of CSII programs (case1: 0.50 U/hour at 0 AM-8 AM, 0.525 U/hour at 8 AM-10 AM, 0.475 U/hour at 10 AM-5 PM and 0.5 U/hour at 5 PM to 12 PM with CIR 8-10-10 g/U; case 2: 0.45 U/hour at 11 PM-1 AM, 0.50 U/hour at 1 AM-5 AM, 0.45 U/hour at 5 AM-10 AM, 0.40 U/hour at 10 AM-7 PM and 0.50 at 7 PM-11 PM with CIR 10-10-9 g/U). We also adjusted the temporary basal rate late before the workup for exercise-induced hypoglycemia.

After that, they continued face-to-face outpatient visits approximately every month and showed decreased HbA1c TBR and increased TAR (Table 1). In particular, they adequately used temporary basal late based on their predicted exercise quantity. Additionally, case 1 was infected with COVID-19 in Japan's seventh wave of the COVID-19 pandemic, nine months after the initiation of CSII. At that time, we again conducted telemedicine follow-up and were able to follow his glucose trends. We advised him to increase basal late via cloud-platform-based isCGM in a remote area.

## Discussion

This report describes two Japanese cases with T1D in whom CSII (MiniMed^TM^ 670G) was successfully initiated using a combination of a video-meeting tool and cloud-platform-based isCGM.

The CGM system has shown significant advances recently. The updated recommendation from the American Diabetes Association [13] noted that MDI or CSII combined with CGM leads to better HbA1c levels and less hypoglycemia in youths and adults with T1D than those without T1D. Insulin delivery systems, including several types of insulin pumps, have been developed over the past few decades. Among them, AID is more expensive than using CSII solely, even with isCGM in Japanese self-funded insurance systems, and some subjects could select CSII (with isCGM) solely instead of AID in Japan. However, CSII is still not widely spread enough in Japan [14], despite its usefulness with isCGM [15]. Insulin pump therapy has mechanical procedure challenges and potential risks to users, resulting in unintended discontinuation of insulin due to kinking of the catheter and inappropriate syringe priming. Therefore, CSII is generally initiated during hospitalization in Japan to provide mechanical trouble education and careful titration of insulin doses [6], unlike other countries introducing it in outpatient settings. However, if subjects live in a place with barriers to healthcare access and elaborate follow-up, initiation of insulin pump therapy becomes difficult, especially during the COVID-19 pandemic. Thus, a consensus has not yet been established regarding selecting appropriate insulin and glucose monitoring systems. Currently, these devices are introduced individually based on socioeconomic status, ethnicity, and educational background [16].

In this case report, we conducted safe and effective CSII initiations using an online meeting tool and the cloud-platform-based isCGM in the subjects residing in a rural area and demonstrated the resulting long-term improvement in HbA1c and TIR similar to the previous report showing CSII advantage in T1D subjects [15]. Similarly, the effectiveness of the online pump training program in introducing the AID has been reported [17,18]. Although these technologies seem very useful and practical in terms of insulin pump education, additional careful follow-up with healthcare professionals is necessary after the initiation of insulin pumps since the mechanism or procedure of insulin pumps is becoming more complicated, and the Food and Drug Administration classifies insulin pumps as class II (moderate-risk) devices [19]. Furthermore, given that the CSII does not have a high- or low-glucose alert system, we should be more careful about the risk of CSII initiation than AID. Under such conditions, a telemedicine approach using a video-based meeting tool and cloud-platform-based isCGM could solve mechanical- or procedure-related glycemic excursions in the early stages after insulin pump initiation in a remote area with barriers to healthcare access. Moreover, the telemedicine approach is reported to improve motivations for glycemic control in subjects with diabetes in remote areas from hospitals [9,20].

Additionally, the chances of using telemedicine are increasing due to the COVID-19 pandemic. Telemedicine with cloud-platform-based isCGM shows efficacy and safety outcomes equal to face-to-face outpatient settings in T1D subjects [10]. The usefulness of telemedicine with CGM in different situations for T1D subjects requires further study.

## Conclusions

This report presents unique cases of CSII initiation using an online meeting tool and cloud platform-based isCGM. There are several economic and geographical barriers to introducing insulin pumps, especially in Japan. Notably, during the COVID-19 pandemic, the telemedicine approach for the subjects wondering about the initiation of CSII might be a solid tool to support their safe and effective outpatient CSII introduction. A randomized study revealing the efficacy and safety of telemedicine for the initiation and follow-up of T1D subjects with insulin pumps would be of great interest.
